# Metformin Use Was Associated With Reduced Risk of Incidental Sjögren's Syndrome in Patients With Type 2 Diabetes: A Population-Based Cohort Study

**DOI:** 10.3389/fmed.2021.796615

**Published:** 2022-01-12

**Authors:** Cheng-You Wang, Jung-Nien Lai, Chin-Hsiu Liu, Kai-Chieh Hu, Kai-Lun Sheu, James Cheng-Chung Wei

**Affiliations:** ^1^Department of Medicine, E-DA Hospital, Kaohsiung, Taiwan; ^2^School of Medicine, Chung Shan Medical University, Taichung, Taiwan; ^3^School of Chinese Medicine, College of Chinese Medicine, China Medical University, Taichung, Taiwan; ^4^Department of Chinese Medicine, China Medical University Hospital, Taichung, Taiwan; ^5^Rheumatology and Immunology Center, China Medical University Hospital, Taichung, Taiwan; ^6^College of Medicine, China Medical University, Taichung, Taiwan; ^7^Management Office for Health Data, China Medical University Hospital, Taichung, Taiwan; ^8^Institute of Medicine, Chung Shan Medical University, Taichung, Taiwan; ^9^Department of Family and Community Medicine, Chung Shan Medical University Hospital, Taichung, Taiwan; ^10^Department of Geriatric Medicine, Chung Shan Medical University Hospital, Taichung, Taiwan; ^11^Graduate Institute of Integrated Medicine, China Medical University, Taichung, Taiwan; ^12^Department of Rheumatology and Immunology, Chung Shan Medical University Hospital, Taichung, Taiwan

**Keywords:** metformin, Sjögren's syndrome, retrospective, cohort, National Health Insurance Research Database (NHIRD)

## Abstract

**Purpose:** Previous studies have shown that metformin exhibits an anti-inflammatory effect and may decrease the risk of incidental diabetes. But the effect of metformin on incidental Sjögren's syndrome is unknown. The aim of the study was to examine the association between metformin exposure and Sjögren's syndrome in diabetic patients.

**Methods:** The dataset in this retrospective cohort study was obtained from the National Health Insurance Research Database (2000–2013) in Taiwan. In total, 15,098 type 2 diabetic patients under metformin treatment and an equivalent number without metformin treatment matched for comparison were included. The primary endpoint was the incidence of Sjogren's syndrome. Univariate and multivariate Cox proportional hazards models were used for data analysis. A subgroup analysis and sensitivity test were also performed.

**Results:** The incidence rate of Sjögren's syndrome in non-metformin controls was 40.83 per 100,000 person-years and 16.82 per 100,000 person-years in metformin users. The adjusted hazard ratio (aHR) in diabetic patients under metformin treatment was 0.46 (95% CI, 0.23 to 0.92). In subgroup analysis, men had a lower risk of developing Sjögren's syndrome than women [aHR = 0.15, 95% CI = (0.05, 0.41)]. After prescribing metformin to type 2 diabetic patients aged 60 years or more, those patients had a lower risk of developing Sjögren's syndrome [aHR = 0.34, 95% CI = (0.12, 0.96)].

**Conclusion:** In this large population-based cohort study, metformin exposure was associated with a reduced risk of developing Sjögren's syndrome in type 2 diabetic patients.

## Introduction

Sjögren's syndrome (SS) is a chronic systemic autoimmune disorder characterized by lymphocytic infiltrates of the affected exocrine gland with various manifestations (1). In addition to dry eye as the most common symptom affecting more than 95% of SS patients, sleep disturbance, dysphagia, oral candidiasis, joint inflammatory, and neurological and multi-organ manifestation have also been reported (2–6). The global prevalence of SS is about 0.2% in the adult population with a male/female ratio of 1:9 according to the classification criteria of the American-European Consensus Group (AECG) (7). Numerous factors, including genes, environment, viruses, and hormones might trigger the progression of the disease mediated particularly by T and B lymphocytes (8–10). Elevated B-cell activating factor (BAFF) level plays an especially important role in the maturation of irregular B cells in exocrine glands (11). Due to the aggravating symptoms and non-negligible life-threatening comorbidities, many studies have been dedicated to developing effective treatments. However, traditional immunosuppressives which are effective in other autoimmune diseases seem to be an unsuccessful therapeutic strategy in SS (12). Numerous biological agents such as rituximab, belimumab, and abatacept have been reported to be effective in patients with SS, except TNF inhibitors (13).

Metformin, an oral anti-hyperglycemic agent, is a first-line therapy for type 2 diabetes mellitus (DM) due to an improvement of insulin sensitivity and a decrease in glucose production (14). Furthermore, survival benefits associated with metformin use in numerous types of cancer have been reported, including colorectal cancer, neck cancer, and non-small-cell lung cancer (15–17). Recently, metformin has shown a new benefit in autoimmune diseases due to its anti-inflammatory and immune-modulatory mechanisms (18). Although several studies have investigated the association between metformin and autoimmune disease, few studies have focused on the impact of metformin on SS. Accordingly, we aimed to investigate whether metformin would be beneficial in reduction of SS in type 2 diabetic patients in the nationwide retrospective cohort study by using the National Health Insurance Research Database (NHIRD).

## Materials and Methods

### Data Source

This study used the Longitudinal Health Insurance Database (LHID), which was randomly sampled from the National Health Insurance Research Database (NHIRD) derived from a single-payer system for healthcare launched in Taiwan in 1995, and 99.9% of Taiwan's population was enrolled. The LHID was released with anonymous and encrypted identifiers for preserving privacy and consisted of comprehensive medical records of one million beneficiaries involving diagnoses of diseases, inpatient and outpatient services, and details of the use of prescription drugs, operations and investigations. The International Classification of Diseases, 9th Revision, Clinical Modification (ICD-9-CM) was used in assigning codes to diagnoses. The study was approved by the Research Ethics Committee at the China Medical University and Hospital [CMUH104-REC2-115(CR-6)].

### Study Population

**Figure 1:**
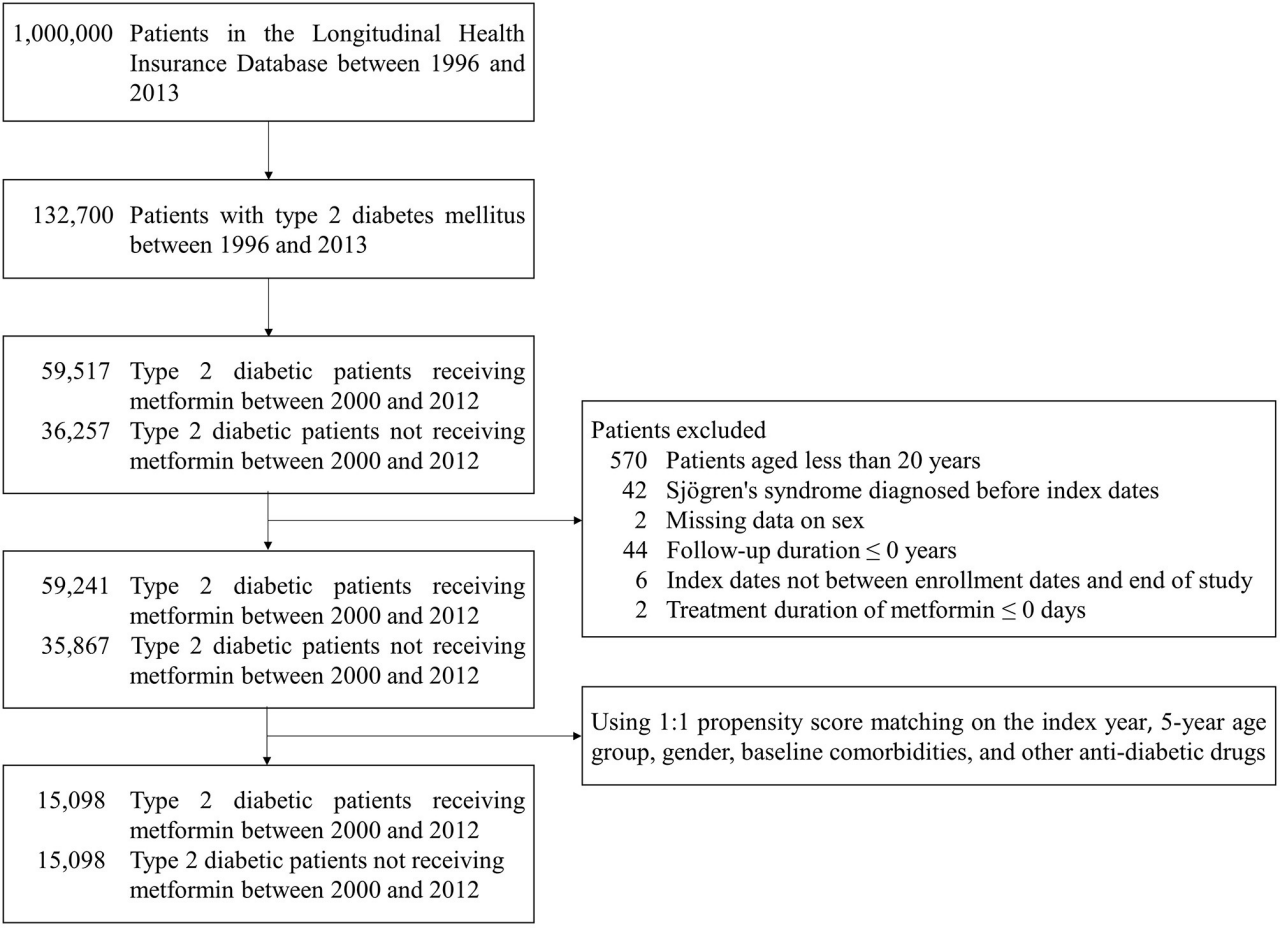
Flowchart of the study population selection.

Patients with at least one inpatient or two outpatient claims of type 2 diabetes mellitus (ICD-9-CM: 250 except 250.x1 and 250.x3) were enrolled (19). Patients who received metformin after the diagnosis of type 2 diabetes mellitus were assigned to the case cohort, and the index date was defined as the date when patients were first prescribed metformin between 2000 and 2012. Type 2 diabetes mellitus patients without metformin treatment were randomly selected and matched with metformin users for the index year, 5-year age group, gender, baseline comorbidities, and other anti-diabetic drugs in a ratio of 1:1 by propensity score matching. The end date of follow-up was the onset date of Sjögren's syndrome (ICD-9-CM: 710.2), the date of withdrawal or death, or December 31st, 2013 (20, 21). The study excluded (1) patients aged <20 years, (2) patients who were diagnosed with Sjögren's syndrome before index dates, (3) patients who had missing data on gender, (4) patients whose follow-up duration was 0 or less, (5) patients whose index dates were not between enrollment dates and end of study, and (6) metformin users whose treatment duration was 0 or less. Figure 1 displays the flowchart of the study population selection.

### Comorbidities and Medications

Baseline comorbidities considered included cirrhosis (ICD-9-CM: 571), hypertension (ICD-9-CM: 401–405), hyperlipidemia (ICD-9-CM: 272.0–272.4), asthma (ICD-9-CM: 493), chronic obstruction pulmonary disease (ICD-9-CM: 490–496), coronary artery disease (ICD-9-CM: 410–414), anxiety (ICD-9-CM: 300), alcohol-related disorders (ICD-9-CM: 291, 303), tobacco use disorder (ICD-9-CM: 305.1), and autoimmune diseases (ICD-9-CM: 710.0, 710.1, 710.3, 710.4, 714.0, 714.30, 714.31, 714.32, 714.33). Patients diagnosed with the comorbidities should have at least one inpatient or two outpatient claims. Other anti-diabetic drugs considered included DPP-4 inhibitors, sulfonylureas, thiazolidinediones, alpha-glucosidase inhibitors, and insulin. All medications should be prescribed after the diagnosis of type 2 diabetes mellitus.

### Statistical Analysis

Descriptive statistics of demographics, comorbidities, and medications were summarized by counts and percentages for the categorical variables and means and standard deviations (SDs) for the continuous variables. The distributions of demographic, comorbidities, and medications between the case and comparison cohorts were compared using standardized mean differences (SMDs). When a SMD was <0.1 in an absolute value, a negligible difference between the two cohorts for the variables was identified. Cumulative incidence rates of events were calculated based on the Kaplan-Meier method, and the log-rank test was used to compare the difference in time-to-event distributions between the case and comparison cohorts. Hazard ratios (HRs) with 95% confidence intervals (95% CIs) were estimated using univariate Cox proportional hazards models; adjusted hazard ratios (aHRs) with 95% CIs were estimated by multivariate Cox proportional hazards models with the covariates of age, gender, comorbidities, and medications. Significant levels of 0.05 were used. To test the proportional assumption for the multivariate Cox regression model, a Wald chi-squared test was performed. Data were analyzed using SAS 9.4 software (SAS Institute Inc., Cary, NC).

## Results

### The Characteristics of the Participants With and Without Metformin Use

Table 1 shows the demographic characteristics, comorbidities and other anti-diabetic drugs in the propensity score matched cohorts with and without metformin among type 2 diabetic patients. The average age of metformin users was 61.54 ± 14.51 years, and males accounted for 50.43% of the users. In the profiles of baseline comorbidities and medications, there were no obvious differences between non-metformin and metformin users.

**Table 1:** Demographic characteristics, comorbidities, and other anti-diabetic drugs in type 2 diabetic patients with and without metformin.

**Variable**	**Total**	**Non-metformin**	**Metformin**	**SMD^**§**^**
	***N* = 30,196**	***N* = 15,098**	***N* = 15,098**	
	***n***	***n* (%)/Mean ± SD**	***n* (%)/Mean ± SD**	
**Age (year)**				
<40	2,158	1,020 (6.76)	1,138 (7.54)	0.0303
40–49	4,155	2,059 (13.64)	2,096 (13.88)	0.0071
50–59	7,446	3,717 (24.62)	3,729 (24.70)	0.0018
≥60	16,437	8,302 (54.99)	8,135 (53.88)	0.0222
Mean ± SD		62.10 ± 14.51	61.54 ± 14.51	0.0387
**Sex**				
Male	15,379	7,765 (51.43)	7,614 (50.43)	0.0200
**Baseline comorbidities**				
Cirrhosis	12,395	6,123 (40.56)	6,272 (41.54)	0.0201
Hypertension	20,013	10,091 (66.84)	9,922 (65.72)	0.0237
Hyperlipidemia	14,383	7,140 (47.29)	7,243 (47.97)	0.0137
Asthma	4,891	2,407 (15.94)	2,484 (16.45)	0.0138
COPD	11,508	5,753 (38.10)	5,755 (38.12)	0.0003
CAD	9,882	4,952 (32.80)	4,930 (32.65)	0.0031
Anxiety	9,426	4,694 (31.09)	4,732 (31.34)	0.0054
Alcohol-related disorders	365	183 (1.21)	182 (1.21)	0.0006
Tobacco use disorder	344	171 (1.13)	173 (1.15)	0.0012
Autoimmune diseases	90	44 (0.29)	46 (0.30)	0.0024
**Other anti-diabetic drugs**				
DPP-4 inhibitors	1,018	467 (3.09)	551 (3.65)	0.0308
Sulfonylureas	11,287	5,756 (38.12)	5,531 (36.63)	0.0308
Thiazolidinediones	1,038	498 (3.30)	540 (3.58)	0.0153
α-glucosidase inhibitors	2,258	1,065 (7.05)	1,193 (7.90)	0.0322
**Follow-up duration (year)**	7,213	3,513 (23.27)	3,700 (24.51)	0.0291

§ *A standardized mean difference of ≤0.1 indicates a negligible difference between the two cohorts*.

*CAD, Coronary Artery Disease; COPD, Chronic Obstruction Pulmonary Disease; PP-4, DiPeptidyl Peptidase-4; SD, Standard Deviation; SMD, Standardized Mean Difference*.

### Risk Factors Associated With Sjögren's Syndrome in Type 2 Diabetic Patients

Table 2 shows Cox regression analyses of Sjögren's syndrome associated with metformin, demographics, baseline comorbidities, and other anti-diabetic drugs in type 2 diabetic patients. The incidence rate of Sjögren's syndrome in non-metformin users was 40.83 per 100,000 person-years; and the incidence rate of Sjögren's syndrome in metformin users was 16.82 per 100,000 person-years. It was shown that metformin could reduce the risk of Sjögren's syndrome in type 2 diabetic patients [aHR = 0.46, 95% CI = (0.23, 0.92)]. When compared to women, men had a lower risk of developing Sjögren's syndrome [aHR = 0.15, 95% CI = (0.05, 0.41)]. In the baseline comorbidities, type 2 diabetic patients with cirrhosis were at a higher risk of developing Sjögren's syndrome [aHR = 2.26, 95% CI = (1.13, 4.54)].

**Table 2:** Cox regression analyses of Sjögren's syndrome associated with metformin, demographics, baseline comorbidities, and other anti-diabetic drugs in type 2 diabetic patients.

**Variable**	**Event**	**Person-year**	**IR**	**Crude**		Adjusted^*^	
	***N* = 36**		**100,000 person-years**	**HR (95% CI)**	***P*-value**	**HR (95% CI)**	***P*-value**
**Metformin**							
No	24	58,779	40.83	1 (Reference)		1 (Reference)	
Yes	12	71,362	16.82	0.43 (0.21, 0.86)	0.0170	0.46 (0.23, 0.92)	0.0292
**Age (year)**							
<40	1	11,756	8.51	1 (Reference)		1 (Reference)	
40–49	6	20,154	29.77	3.38 (0.41, 28.10)	0.2593	4.40 (0.52, 37.03)	0.1729
50–59	11	32,639	33.70	3.73 (0.48, 28.94)	0.2077	4.74 (0.59, 38.03)	0.1427
≥60	18	65,591	27.44	2.99 (0.40, 22.43)	0.2866	4.83 (0.60, 39.12)	0.1400
**Sex**							
Female	32	66,541	48.09	1 (Reference)		1 (Reference)	
Male	4	63,599	6.29	0.13 (0.05, 0.36)	0.0001	0.15 (0.05, 0.41)	0.0003
**Baseline comorbidities**							
**Cirrhosis**							
No	14	76,085	18.40	1 (Reference)		1 (Reference)	
Yes	22	54,055	40.70	2.22 (1.13, 4.34)	0.0198	2.26 (1.13, 4.54)	0.0214
**Hypertension**							
No	18	49,206	36.58	1 (Reference)		1 (Reference)	
Yes	18	80,935	22.24	0.58 (0.30, 1.11)	0.0994	0.47 (0.22, 1.00)	0.0511
**Hyperlipidemia**							
No	17	70,369	24.16	1 (Reference)		1 (Reference)	
Yes	19	59,772	31.79	1.27 (0.66, 2.46)	0.4730	0.94 (0.47, 1.90)	0.8711
**Asthma**							
No	28	111,701	25.07	1 (Reference)		1 (Reference)	
Yes	8	18,440	43.38	1.67 (0.76, 3.66)	0.2026	1.16 (0.45, 3.02)	0.7565
**COPD**							
No	19	84,762	22.42	1 (Reference)		1 (Reference)	
Yes	17	45,379	37.46	1.62 (0.84, 3.12)	0.1505	1.39 (0.61, 3.15)	0.4365
**CAD**							
No	24	90,538	26.51	1 (Reference)		1 (Reference)	
Yes	12	39,602	30.30	1.11 (0.56, 2.23)	0.7625	1.03 (0.46, 2.29)	0.9421
**Anxiety**							
No	19	90,705	20.95	1 (Reference)		1 (Reference)	
Yes	17	39,436	43.11	2.03 (1.05, 3.90)	0.0345	1.30 (0.64, 2.62)	0.4631
**Alcohol-related disorders**							
No	36	128,831	27.94	1 (Reference)		1 (Reference)	
Yes	0	1,310	0.00	NA	NA	NA	NA
**Tobacco use disorder**							
No	36	129,097	27.89	1 (Reference)		1 (Reference)	
Yes	0	1,043	0.00	NA	NA	NA	NA
**Autoimmune diseases**							
No	36	129,797	27.74	1 (Reference)		1 (Reference)	
Yes	0	344	0.00	NA	NA	NA	NA
**Other anti-diabetic drugs**							
**DPP-4 inhibitors**							
No	36	124,956	28.81	1 (Reference)		1 (Reference)	
Yes	0	5,185	0.00	NA	NA	NA	NA
**Sulfonylureas**							
No	29	78,062	37.15	1 (Reference)		1 (Reference)	
Yes	7	52,079	13.44	0.38 (0.16, 0.86)	0.0204	0.51 (0.21, 1.22)	0.1297
**Thiazolidinediones**							
No	36	124,403	28.94	1 (Reference)		1 (Reference)	
Yes	0	5,738	0.00	NA	NA	NA	NA
**α-glucosidase inhibitors**							
No	32	119,006	26.89	1 (Reference)		1 (Reference)	
Yes	4	11,135	35.92	1.39 (0.49, 3.94)	0.5329	1.80 (0.63, 5.14)	0.2752
**Insulin**							
No	33	101,450	32.53	1 (Reference)		1 (Reference)	
Yes	3	28,690	10.46	0.32 (0.10, 1.05)	0.0599	0.52 (0.15, 1.80)	0.3010

*CAD, Coronary Artery Disease; CI, Confidence Interval; COPD, Chronic Obstruction Pulmonary Disease; DPP-4, DiPeptidyl Peptidase-4; HR, Hazard Ratio; IR, Incidence Rate*.

* *Adjusted for demographics, baseline comorbidities, and other anti-diabetic drugs listed above*.

### Stratification Analysis of Type 2 Diabetic Patients With and Without Metformin

Table 3 shows comparison of incidence of Sjögren's syndrome in type 2 diabetic patients with and without metformin stratified by demographics, baseline comorbidities, and other anti-diabetic drugs. After prescribing metformin to type 2 diabetic patients aged 60 years or more, those patients had a lower risk of developing Sjögren's syndrome [aHR = 0.34, 95% CI = (0.12, 0.96)].

**Table 3:** Comparisons of incidence of Sjögren's syndrome in type 2 diabetic patients with and without metformin stratified by demographics, baseline comorbidities, and other anti-diabetic drugs.

**Variable**	**Non-metformin**			**Metformin**			**Metformin vs. non-metformin**			
	**Event**	**Person-year**	**IR**	**Event**	**Person-year**	**IR**	**Crude**		Adjusted^*^	
	***N* = 24**		**100,000 person-years**	***N* = 12**		**100,000 person-years**	**HR (95% CI)**	***P*-value**	**HR (95% CI)**	***P*-value**
All	24	58,779	40.83	12	71,362	16.82	0.43 (0.21, 0.86)	0.0170	0.46 (0.23, 0.92)	0.0292
**Age (year)**										
20–39	1	5,314	18.82	0	6,442	0.00	NA		NA	
40–59	3	9,326	32.17	3	10,828	27.71	0.79 (0.16, 3.94)	0.7744	0.90 (0.17, 4.67)	0.8998
50–59	7	15,189	46.09	4	17,450	22.92	0.51 (0.15, 1.74)	0.2805	0.58 (0.17, 2.03)	0.3982
≥60	13	28,949	44.91	5	36,642	13.65	0.34 (0.12, 0.96)	0.0423	0.34 (0.12, 0.96)	0.0407
**Sex**										
Female	20	30,255	66.11	12	36,287	33.07	0.53 (0.26, 1.08)	0.0782	0.57 (0.28, 1.17)	0.1249
Male	4	28,524	14.02	0	35,075	0.00	NA	NA	NA	NA
**Baseline comorbidities**										
**Cirrhosis**										
No	11	34,373	32.00	3	41,713	7.19	0.24 (0.07, 0.88)	0.0305	0.27 (0.07, 0.96)	0.0434
Yes	13	24,406	53.27	9	29,649	30.35	0.58 (0.25, 1.36)	0.2079	0.61 (0.26, 1.44)	0.2607
**Hypertension**										
No	12	22,131	54.22	6	27,075	22.16	0.41 (0.15, 1.09)	0.0725	0.48 (0.18, 1.30)	0.1495
Yes	12	36,647	32.74	6	44,287	13.55	0.46 (0.17, 1.23)	0.1232	0.46 (0.17, 1.24)	0.1241
**Hyperlipidemia**										
No	11	30,750	35.77	6	39,619	15.14	0.46 (0.17, 1.25)	0.1299	0.48 (0.18, 1.30)	0.1497
Yes	13	28,029	46.38	6	31,743	18.90	0.41 (0.16, 1.08)	0.0725	0.47 (0.18, 1.25)	0.1291
**Asthma**										
No	19	50,598	37.55	9	61,103	14.73	0.40 (0.18, 0.89)	0.0254	0.43 (0.19, 0.97)	0.0415
Yes	5	8,180	61.12	3	10,259	29.24	0.52 (0.12, 2.16)	0.3652	0.55 (0.13, 2.33)	0.4213
**COPD**										
No	13	38,222	34.01	6	46,540	12.89	0.40 (0.15, 1.05)	0.0635	0.47 (0.18, 1.24)	0.1260
Yes	11	20,557	53.51	6	24,822	24.17	0.46 (0.17, 1.25)	0.1272	0.47 (0.17, 1.27)	0.1342
**CAD**										
No	15	40,991	36.59	9	49,548	18.16	0.51 (0.22, 1.17)	0.1102	0.57 (0.25, 1.30)	0.1794
Yes	9	17,788	50.60	3	21,814	13.75	0.29 (0.08, 1.08)	0.0656	0.29 (0.08, 1.07)	0.0638
**Anxiety**										
No	12	40,340	29.75	7	50,365	13.90	0.50 (0.19, 1.26)	0.1415	0.52 (0.20, 1.32)	0.1668
Yes	12	18,438	65.08	5	20,997	23.81	0.37 (0.13, 1.06)	0.0646	0.41 (0.14, 1.18)	0.0987
**Alcohol-related disorders**										
No	24	58,195	41.24	12	70,636	16.99	0.43 (0.21, 0.86)	0.0170	0.46 (0.23, 0.92)	0.0292
Yes	0	584	0.00	0	726	0.00	NA	NA	NA	NA
**Tobacco use disorder**										
No	24	58,260	41.19	12	70,838	16.94	0.43 (0.21, 0.86)	0.0169	0.46 (0.23, 0.92)	0.0292
Yes	0	519	0.00	0	524	0.00	NA	NA	NA	NA
**Autoimmune diseases**										
No	24	58,613	40.95	12	71,184	16.86	0.43 (0.21, 0.86)	0.0169	0.46 (0.23, 0.92)	0.0292
Yes	0	165	0.00	0	179	0.00	NA	NA	NA	NA
**Other anti-diabetic drugs**										
**DPP-4 inhibitors**										
No	24	56,626	42.38	12	68,330	17.56	0.43 (0.22, 0.86)	0.0176	0.46 (0.23, 0.92)	0.0292
Yes	0	2,153	0.00	0	3,032	0.00	NA	NA	NA	NA
**Sulfonylureas**										
No	20	37,290	53.63	9	40,772	22.07	0.42 (0.19, 0.91)	0.0285	0.44 (0.20, 0.97)	0.0425
Yes	4	21,489	18.61	3	30,590	9.81	0.59 (0.13, 2.66)	0.4928	0.61 (0.13, 2.75)	0.5178
**Thiazolidinediones**										
No	24	56,583	42.42	12	67,819	17.69	0.43 (0.22, 0.87)	0.0181	0.46 (0.23, 0.92)	0.0292
Yes	0	2,195	0.00	0	3,543	0.00	NA	NA	NA	NA
**α-glucosidase inhibitors**										
No	22	54,368	40.47	10	64,638	15.47	0.40 (0.19, 0.84)	0.0154	0.45 (0.21, 0.95)	0.0361
Yes	2	4,411	45.34	2	6,724	29.75	0.73 (0.10, 5.19)	0.7500	0.34 (0.03, 3.87)	0.3869
**Insulin**										
No	22	47,999	45.83	11	53,452	20.58	0.46 (0.22, 0.95)	0.0360	0.49 (0.24, 1.02)	0.0559
Yes	2	10,780	18.55	1	17,910	5.58	0.33 (0.03, 3.70)	0.3701	0.28 (0.02, 4.10)	0.3520

*CAD, Coronary Artery Disease; CI, Confidence Interval; COPD, Chronic Obstruction Pulmonary Disease; DPP-4, DiPeptidyl Peptidase-4; HR, Hazard Ratio; IR, Incidence Rate*.

* *Adjusted for demographics, baseline comorbidities, and other anti-diabetic drugs listed above*.

Table 4 shows Cox regression analyses of Sjögren's syndrome associated with different treatment duration and cumulative doses of metformin in type 2 diabetic patients. When treatment duration of metformin was 90 days or more, the risk of Sjögren's syndrome decreased in type 2 diabetic patients with metformin in contrast to those without metformin [aHR = 0.27, 95% CI = (0.10, 0.71)]. On the other hand, when cumulative doses of metformin was 45,000 mg or more, the risk of Sjögren's syndrome also decreased in type 2 diabetic patients with metformin in contrast to those without metformin [aHR = 0.30, 95% CI = (0.12, 0.74)].

**Table 4:** Cox regression analyses of Sjögren's syndrome associated with different treatment duration and cumulative doses of metformin in type 2 diabetic patients.

**Variable**	**Event**	**Person-year**	**IR**	**Crude**		Adjusted^*^	
	***N* = 36**		**100,000 person-years**	**HR (95% CI)**	***P*-value**	**HR (95% CI)**	***P*-value**
**Treatment duration (day)**							
None	24	58,779	40.83	1 (Reference)		1 (Reference)	
1–29	3	7,603	39.46	0.99 (0.30, 3.28)	0.9809	0.85 (0.25, 2.85)	0.7937
30–89	4	9,141	43.76	1.09 (0.38, 3.15)	0.8683	1.00 (0.35, 2.91)	0.9963
≥ 90	5	54,618	9.15	0.24 (0.09, 0.62)	0.0033	0.27 (0.10, 0.71)	0.0077
**Cumulative doses (mg)**							
None	24	58,779	40.83	1 (Reference)		1 (Reference)	
1–14,999	3	6,332	47.38	1.17 (0.35, 3.89)	0.7980	0.99 (0.30, 3.32)	0.9914
15,000–44,999	3	7,089	42.32	1.06 (0.32, 3.51)	0.9281	0.96 (0.29, 3.21)	0.9490
≥45,000	6	57,941	10.36	0.27 (0.11, 0.65)	0.0037	0.30 (0.12, 0.74)	0.0087

*CI, Confidence Interval; HR, Hazard Ratio; IR, Incidence Rate*.

* *Adjusted for demographics, baseline comorbidities, and medications listed above*.

### Long-Term Trends in Metformin Use and the Risk of Sjögren's Syndrome

**Figure 2:**
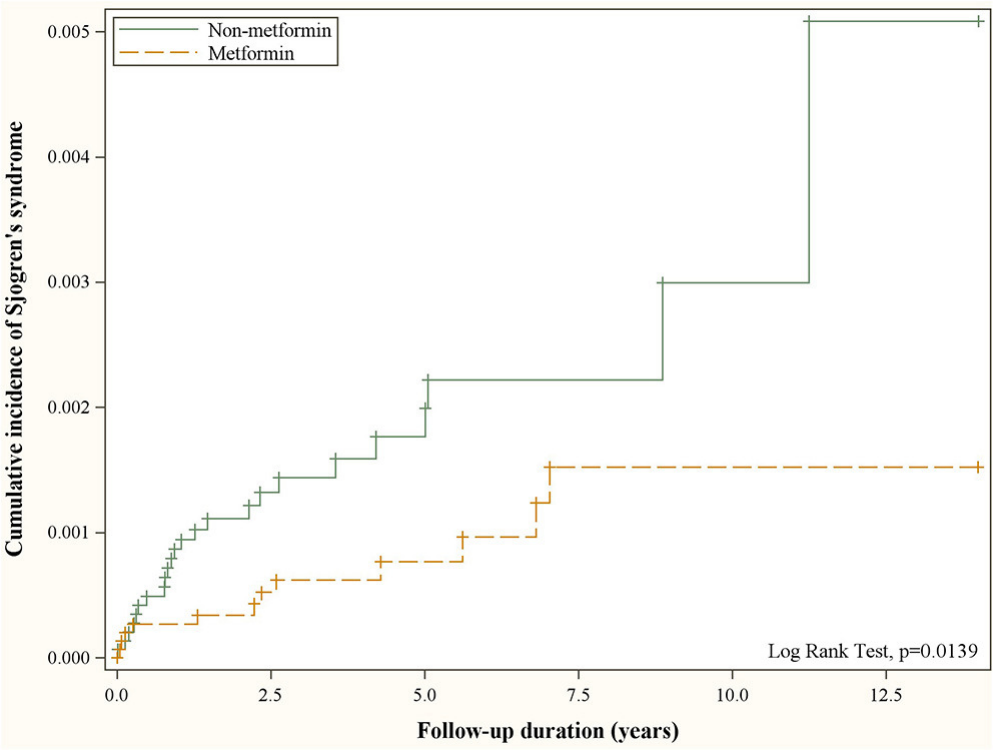
Cumulative incidence of Sjögren's syndrome in patients with and without metformin use obtained using the Kaplan–Meier method.

Figure 2 depicts the cumulative incidence of Sjögren's syndrome curves in type 2 diabetic patients with and without metformin. The resulting *p*-value for the log rank test between the curves of two cohorts was <0.05, and the case cohort was more likely to have a lower risk of developing Sjögren's syndrome than the comparison cohort.

### Testing the Proportional Hazard Assumption in the Multivariate Cox Model

Table 5 shows the proportionality assumption test for the multivariate Cox regression model in Table 2. We generated the time dependent covariates by creating interactions of the predictors and a natural logarithmic function of follow-up duration and included these in the multivariate model used in Table 2. The result shows that we could not reject the proportionality assumption in the case (*p*-value = 0.3679).

**Table 5:** Test of proportional hazard assumption for the multivariate Cox regression model used in Table 2.

**Parameter**	**Wald Chi-Square**	**DF**	***P*-value**
Proportionality Test	18.3363	17	0.3679

*DF, Degrees of Freedom*.

## Discussion

In this nationwide population-based cohort study, we found that diabetic patients exposed to metformin had a reduced risk of SS compared to those without metformin use [aHR = 0.46, 95% CI = (0.23, 0.92)]. In subgroup analysis, type 2 diabetic patients aged 60 years or more had a lower risk of developing Sjögren's syndrome under metformin use [aHR = 0.34, 95% CI = (0.12, 0.96)].

Several studies have revealed the novel use of metformin for its anti-inflammatory and immune-modulatory effects (22). Recently, distinct benefits between various autoimmune diseases and the use of metformin have been noted in observational cohort studies of psoriasis, multiple sclerosis, myasthenia gravis, ankylosing spondylitis, and rheumatoid arthritis (RA) (23–26). Metformin may also reduce all-cause mortality and admission rate among patients with autoimmune disease (27). Data from several animal models, including experimental autoimmune encephalomyelitis, collagen antibody-induced arthritis, inflammatory bowel disease and Roquin^san/san^ model of systemic lupus erythematosus, strongly supported the immune-modulatory effect of metformin with abilities to suppress T helper (Th)17 cells, promote regulatory T (Treg) cells production, or reduce autoreactive marginal B cells and geminal center formation (28, 29). At the molecular level, these immuno-modulatory effects of metformin were characterized by an increased activation of 5'-AMP-activated protein kinase (AMPK) with subsequent decrease in phosphorylation of mammalian target of rapamycin (mTOR) and the signal transducer and activator of transcription (STAT) 3 pathway. Another study also demonstrated that metformin could inhibit the proliferation of human RA-fibrobalst-like synoviocytes through cell cycle arrest by regulating the insulin-like growth factor receptor/phosphoinositide kinase 3/ protein kinase B/ m-TOR pathway (30). Moreover, the indirect effects of metformin on anti-inflammation might also be induced by an improvement in hyperglycemic episodes, weight reduction, and lipid control after its prescription (31). Our longitudinal population-based study provides strong evidence of the reduced risk of SS in metformin-treated patients with type 2 diabetes mellitus. Although few large-scale studies have investigated the new use of this old drug in SS, there are two available studies that support our results (32, 33). A significant decrease in the ratio of Th17/Treg cells in peripheral blood with the improvement of clinical symptoms was observed in patients with SS after metformin treatment (32). In addition, a murine model of SS revealed that metformin could ameliorate salivary gland inflammation by downregulating the expression of interleukin (IL)-6, tumor necrosis factor-α, and IL-17 *in situ*, maintaining the balance between effector T and Treg cells and controlling B cells differentiation (33).

In our study, medications including other anti-diabetic drugs between the case and comparison cohorts were compared. Dipeptidyl peptidase-4 inhibitors (DPP4i), Sulfonylureas, Thiazolidinediones, α-glucosidase and insulin may not reduce the incidence of SS. However, a retrospective cohort study showed that DPP4i might reduce the incidence of autoimmune disorders in type 2 DM patients with HR 0.56 (95% CI 0.53–0.60; *P* < 0.001) (34). The underlying mechanism might be attributed to the important role of CD26/DPP4 in T cell development and memory T cell generation (35). A case report also showed that the use of gliclazide might induce the insulin autoimmune syndrome (36). Further studies are necessary to investigate the underlying mechanism.

Our study possesses several strengths compared to previous studies. To the best of our knowledge, this is the first worldwide population-based study that revealed the association between metformin and reduced risk of SS. The nationwide database covering >99% of the population avoided selection bias. Furthermore, a previous animal model study supports our hypothesis and correlates well with our current results (33). Second, due to the aggravating symptoms and life-threatening comorbidities of SS, many traditional immunotherapy and biological medicines have been investigated. However, the results of these traditional medicines on SS have not been on a par with the marked efficacy seen in treating other autoimmune diseases such as RA and systemic lupus erythematosus (12). Therefore, our findings alternatively offer a preclinical background that the new use of metformin could be reconsidered in clinical trials designed to prove its efficacy in patients with SS due to its well-established safety profile and low cost.

This study has several limitations. First, the incidence of SS in metformin users, in contrast to non-metformin users, was not significant in the subgroup analysis of age even though the overall aHR was significant. There might be some confounding factors that could affect the risk assessment of SS. Different aspects of individual lifestyle, including smoking, alcohol use, daily diet plan, coexisting autoimmune diseases, were all possible confounders. Second, although the sample size in our study is large (*N* = 30,196), the number of events are very small (*N* = 24). Any misclassification in the outcome variable can easily change the *p*-value of the adjusted hazard ratio to >0.05. Our study focused on SS in the Taiwan database diagnosed between 2000 and 2013. Misclassification bias might have existed due to the modification of the diagnosis criteria in SS; for instance, the inclusion of salivary gland ultrasonography in the 2016 American College of Rheumatology (ACR)/European League Against Rheumatism (EULAR) classification criteria slightly increased the sensitivity from 87.4 to 91.1% (37). Third, the association between the mean daily metformin dose and the risk of SS should be elucidated in further studies. Forth, the manuscript lacked detailed information on patient characteristics at baseline, including past/family history, signs and symptoms of SS, organ manifestation, and concomitant use of treatments.

## Conclusion

In conclusion, this 13-year, nationwide, population-based retrospective study demonstrated that type 2 diabetic patients with metformin treatment is associated with a reduced risk of developing SS. Further studies are required to strengthen the result in clinical trial, and to examine the underlying mechanism.

## Data Availability Statement

The original contributions presented in the study are included in the article/supplementary material, further inquiries can be directed to the corresponding author.

## Author Contributions

C-YW: drafting the work. J-NL: substantial contributions to the conception or design of the work. K-CH: the acquisition, analysis, or interpretation of data for the work. K-LS and C-HL: revising it critically for important intellectual content. JW: providing final revision and agreement to all aspects of this research and manuscript in order to ensure the ultimate accuracy and quality of this work. All authors contributed to the article and approved the submitted version.

## Funding

This study was supported in part by Taiwan Ministry of Health and Welfare Clinical Trial Center (MOHW109-TDU-B-212-114004), MOST Clinical Trial Consortium for Stroke (MOST 109-2321-B-039-002), China Medical University Hospital (DMR-109-231), Tseng-Lien Lin Foundation, Taichung, Taiwan.

## Conflict of Interest

The authors declare that the research was conducted in the absence of any commercial or financial relationships that could be construed as a potential conflict of interest.

## Publisher's Note

All claims expressed in this article are solely those of the authors and do not necessarily represent those of their affiliated organizations, or those of the publisher, the editors and the reviewers. Any product that may be evaluated in this article, or claim that may be made by its manufacturer, is not guaranteed or endorsed by the publisher.
